# Research on Intelligent Monitoring and Protection Equipment of Vital Signs of Underground Personnel in Coal Mines: Review

**DOI:** 10.3390/s25010063

**Published:** 2024-12-25

**Authors:** Yuntao Liang, Yingjie Liu, Changjia Lu, Dawei Cui, Jinghu Yang, Rui Zhou

**Affiliations:** 1 Chinese Institute of Coal Science, Beijing 100013, Chinayjh_cumtb@126.com (J.Y.); 2 Emergency Research Institute, Chinese Institute of Coal Science, Beijing 100013, China; 3 Shenyang Research Institute, China Coal Technology and Engineering Group, Shenyang 113122, China

**Keywords:** coal mine safety, vital signs monitoring, intelligent equipment, environmental monitoring, motion recognition

## Abstract

The coal industry is a high risk, high difficulty industry, and the annual global mine accident rate is high, so the safety of coal mine underground operations has been a concern. With the development of technology, the application of intelligent security technology in coal mine safety has broad prospects. In this paper, the research progress of vital signs monitoring and support equipment for underground personnel in coal mines is reviewed. The two main methods to ensure the safety of miners are discussed. They consist of directly monitoring human vital signs through portable devices such as smart helmets and smartwatches and indirectly monitoring underground environmental parameters. In addition, the application of information technology, sensor technology and artificial intelligence in vital signs monitoring is briefly discussed, and some future research directions are proposed. For example, through big data and artificial intelligence technology, vital signs data can be compared with historical data, individual health trends and potential risks can be analyzed, and we can provide personalized health management programs for miners. These technologies not only improve the safety of underground coal mine operation, but also provide an important guarantee for the realization of intelligent and safe coal mine production.

## 1. Introduction

China, characterized by its resource endowment of “abundant coal, scarce oil, and limited gas”, has become the world’s largest producer and consumer of coal, with coal resources playing a crucial role in the national energy structure. Coal mining is a high-risk and challenging industry. The complex mining environment presents various hazards, such as gas explosions, coal dust explosions, mine fires, roof collapses, and water inrushes, leading to frequent underground accidents that severely threaten miners’ safety. Currently, China has approximately 3000 coal mines in operation, employing over 2.8 million workers. Ensuring the safety of underground workers is fundamental and essential for the healthy development of the coal industry.

With technological advancements, coal mine safety measures have been continuously improved and upgraded. Numerous production accidents have shown a close relationship between accidents and human behavior. Humans are the most dynamic factor in productivity; during the production process, factors such as fatigue, emotional fluctuations, distraction, and physiological indicators significantly affect safety. These factors not only alter human behavior but also adversely impact personal safety. However, traditional safety management methods and technical approaches have limitations when dealing with the complex and variable underground environment. Therefore, using advanced technologies for coal mine safety research, particularly the monitoring and analysis of underground personnel’s vital signs, has significant practical importance [1,2].

President Xi Jinping has emphasized the importance of “putting people first and prioritizing life”. In July 2022, the Ministry of Emergency Management’s National Mine Safety Administration issued the “14th Five-Year Plan for Mine Safety Production”, which introduced new requirements for remote, visualized, and intelligent personnel safety protection equipment, encouraging research on “safety protection technologies and products for on-site personnel”. In April 2024, the National Mine Safety Administration released the “Guiding Opinions on Deepening the Promotion of Mine Intelligence Construction to Enhance Mine Safety Development”, which addressed issues of imbalance, inadequacy, and inconsistency in mine intelligence development by proposing 20 guiding opinions. By 2030, the aim is to establish a comprehensive system of mine intelligence technology, equipment, and management; achieve deep integration and sharing of mine data; promote reduced or unstaffed mining operations; effectively control major safety risks; and significantly improve intrinsic mine safety levels.

Monitoring personnel’s vital signs is a critical aspect of ensuring miners’ safety. Vital signs are the basic indicators of physiological activity, including body temperature, heart rate, respiratory rate, and blood pressure. Monitoring these indicators allows for timely assessment of underground workers’ health status, prediction, and prevention of potential health issues, ensuring they can work in a safe environment. The unique underground environment poses numerous challenges for vital signs monitoring. Factors such as high temperatures, high humidity, dust, and toxic gases deep in the mines can adversely affect the human body. Prolonged, high-intensity physical labor can also lead to fatigue, dehydration, and heatstroke among miners. Traditional vital signs monitoring methods often require professional medical personnel and cannot achieve real-time, dynamic monitoring. Therefore, achieving continuous and reliable monitoring of miners’ vital signs in the complex underground environment is an urgent technical challenge [3].

With the rapid development of information technology, sensing technology, and artificial intelligence, the application prospects of intelligent safety technologies in coal mine safety are broad [4,5]. Smartphones, as the most developed and convenient devices, can already identify some human behaviors by monitoring the owner’s actions [6], but they are difficult to carry during underground operations. This necessitates ensuring miners’ safety through ubiquitous, efficient, and cost-effective wearable intelligent equipment to reduce construction injuries and accidents. Through intelligent sensors, wireless communication, and big data analysis, real-time monitoring and intelligent analysis of underground personnel’s vital signs can be achieved, providing strong support for miners’ safety. The advancement of intelligent sensor technology has made vital signs monitoring devices for temperature, heart rate, and respiratory rate more compact, lightweight, and intelligent. These devices can be worn by miners to continuously collect vital signs data, which can then be transmitted to the surface monitoring center via wireless communication. Combining big data analysis and artificial intelligence algorithms allows for the processing and analysis of the collected data, timely detection of anomalies, and issuance of warnings, thereby achieving intelligent safety protection for underground personnel.

## 2. Direct Monitoring of Vital Signs

**Figure 1:**
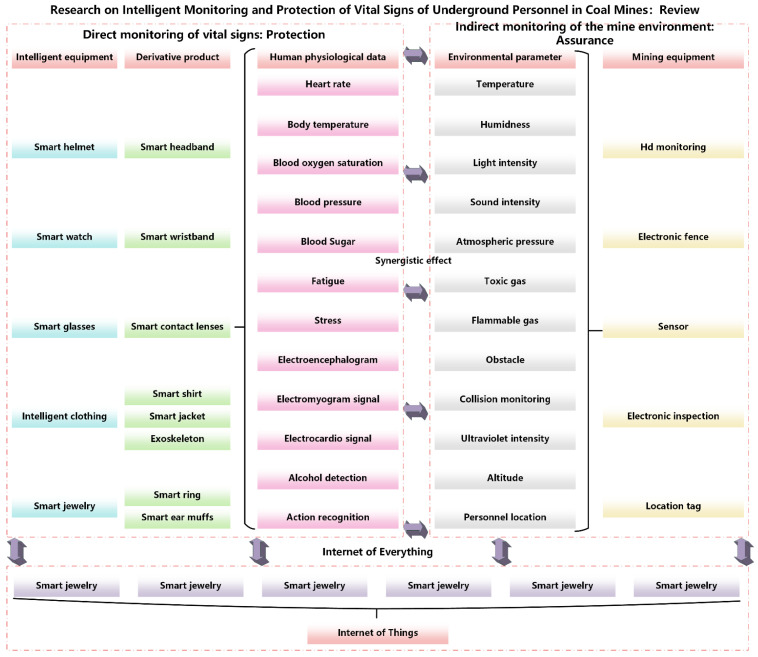
Intelligent equipment summary (different colors represent different levels of things).

All kinds of environmental monitoring techniques and related equipment in coal mine have become increasingly mature, but the research on personnel vital signs monitoring is still mainly concentrated in the medical field, and there are few studies on personnel vital signs monitoring in coal mines. Zinab Abuwarda et al. [7], in 2022, cross-analyzed the application prospects of wearable devices in the medical field in the construction industry, and the widespread use of intelligent equipment greatly improved the safety of construction workers. In order to comprehensively monitor the life safety of coal mine personnel, the monitoring of human vital signs is mainly carried out from two aspects: first, the direct monitoring of human vital signs is mainly through a variety of portable devices, such as helmets and watches, enabling real-time acquisition of miners’ vital signs data. These devices often integrate multiple sensors to continuously monitor key indicators such as body temperature, heart rate, respiratory rate and blood pressure. An important feature of these portable devices is their convenience and efficiency. Miners just need to wear the device, and the sensor can automatically collect data without performing complicated operations. In addition, modern portable devices are often equipped with wireless communication modules that can transmit data in real time to the ground monitoring center for remote monitoring and data analysis. Through big data and artificial intelligence technology, these vital signs data can be compared with historical data to analyze individual health trends and potential risks, so as to provide personalized health management programs. The second is to indirectly monitor the coal mine environment to ensure the safety of human life. The underground environment of coal mine is complex and changeable, and factors such as temperature, humidity, noise and harmful gas concentration may have a great impact on the health of miners. By monitoring these environmental parameters, we can indirectly understand their impact on miners’ vital signs and take corresponding protective measures. The combination of direct monitoring and indirect monitoring can form a comprehensive life safety guarantee system. Direct monitoring, which provides real-time data on miners’ vital signs, and indirect monitoring, which assesses risks from an environmental perspective, complement each other to keep miners safe. Intelligent monitoring equipment and equipment for vital signs of coal mine personnel are shown in Figure 1.

**Figure 2:**
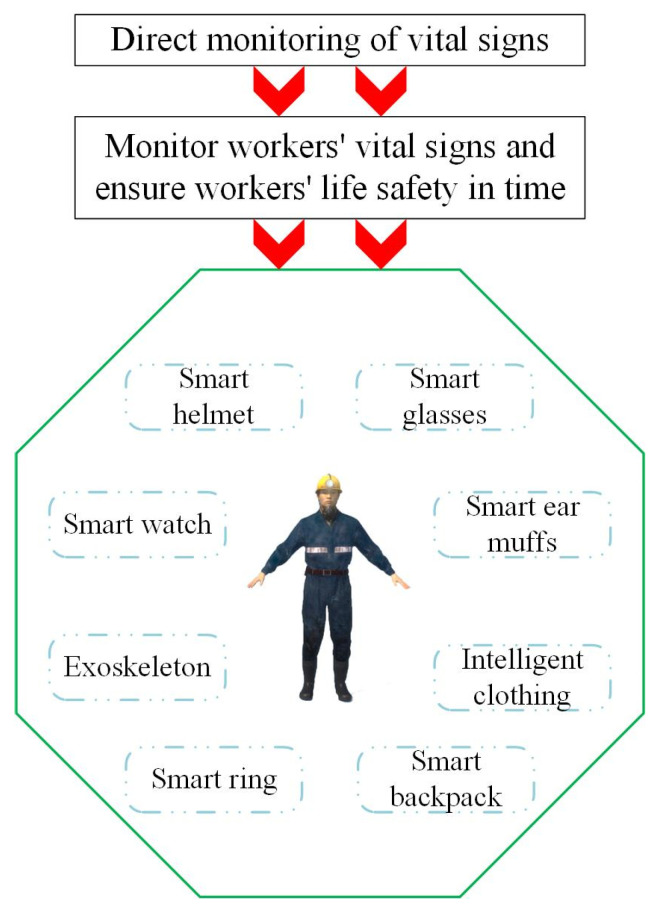
Direct monitoring of vital signs.

Intelligent equipment [8] primarily involves embedding sensors and other miniature electronic devices into traditional wearable items to facilitate convenient monitoring of human physiological characteristics. Currently, this technology has been integrated into various traditional wearables such as helmets, headbands, watches, glasses, hats, and rings. It includes sensors for physiological monitoring, such as EEG (electroencephalogram) monitoring, heart rate monitoring, pulse monitoring, alcohol detection, and fatigue monitoring, as well as sensors for environmental monitoring, such as humidity, temperature, gas, and pressure sensors. The direct monitoring process is illustrated in Figure 2.

### 2.1. Smart Helmet

Due to the unique distribution of coal mining in China, the vast majority of mines are underground. Helmets, as essential equipment for underground workers, have been in use for several decades. However, most of the improvements and upgrades to helmets in China have focused primarily on changes in materials. While these helmets provide some level of protection, they are far from meeting the needs of safe production. Therefore, to better ensure the safety of underground workers, the development of helmets must incorporate more intelligent features.

**Figure 3:**
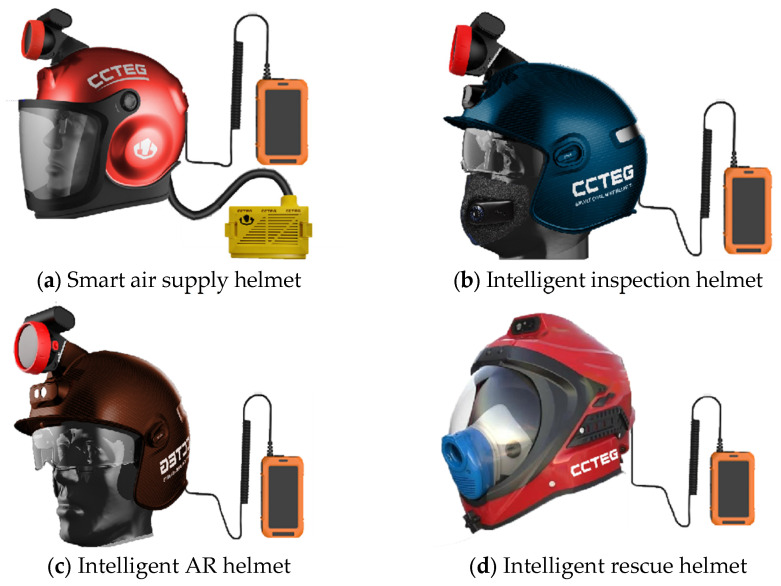
Smart helmets used in coal mines.

The early development of smart helmets abroad, particularly the intelligent design of military helmets in the last century, laid the groundwork for subsequent advancements. Currently, the focus is mainly on upgrading daily helmets, such as those for bicycles and motorcycles. In 2007, Walsh [9] from Ireland developed a smart rugby helmet that could integrate multiple sensors, including accelerometers, temperature sensors, heart rate sensors, and SpO2 sensors, to provide maximum protection against impacts during rugby. Industrial smart helmets have progressed relatively slowly, but the overall research pace remains similar. In 2006, a Japanese company developed the initial industrial smart helmet, which only included a simple integration of communication modules and positioning systems. In the subsequent decade, the development of smart helmets accelerated rapidly. In 2013, the U.S. company Skully launched the Skully AR-1 bicycle helmet, the first widely recognized smart helmet. This helmet featured a transparent head-mounted display, rear-view camera, Bluetooth connectivity, and navigation system, and was acclaimed as the first smart helmet aimed at the consumer market [10]. However, the addition of electronic components, batteries and sensors significantly increased the weight and volume of the helmet, and wearing it for a long time may lead to discomfort or fatigue. Therefore, the helmet (Chinese Institute of Coal Science, Beijing, China) shown in Figure 3 has been further optimized from the material problem of the original helmet, and equipment with lighter materials has been selected to ensure the performance of the helmet. Each individual helmet weighs no more than 2 kg. In addition, different downhole operations require different capabilities of the helmet, as shown in Figure 3. According to the different requirements, the helmet is equipped with different sensors, which greatly ensures that the weight of the helmet itself will not have a negative impact on the operator. At the same time, the helmet is specific, so that it faces the characteristics of each working scene in a coal mine and has different roles. For example, the individual soldier intelligent AR helmet shown in Figure 3c incorporates the design of AR glasses. On the one hand, the external undertaking camera device ensures that the operator can see the scene more clearly in a dim environment. On the other hand, when the operators carry out a fixed work process, the algorithm guides the operators in the underground fixed positions, thereby reducing the human error rate and ensuring the smooth progress of the work. 

**Figure 4:**
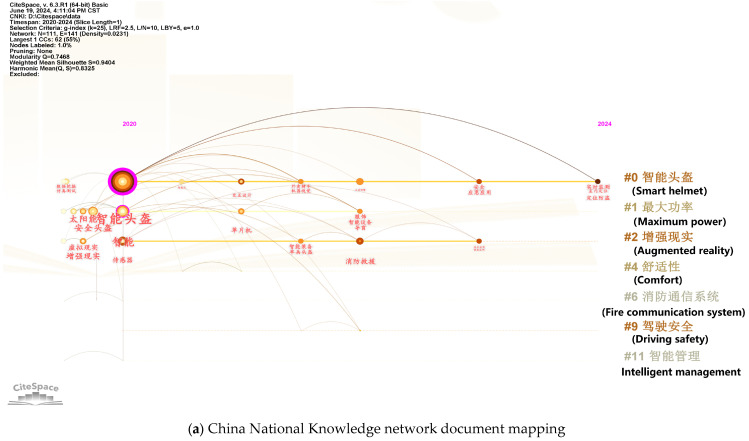

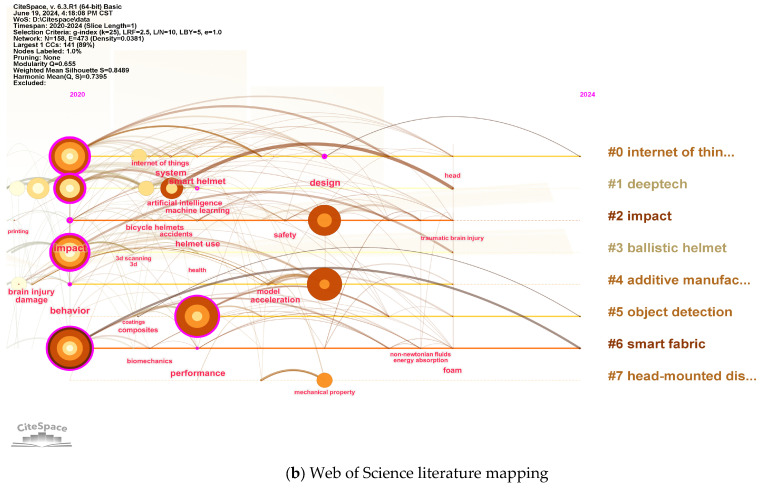
Plot according to the timeline with smart helmet as the keyword.

According to data from Yosoon Choi [11], the average number of published articles on related smart helmets was 1.28 per year before 2015. In 2016 and 2017, this number surged to 8.5 articles per year, and continued to rise annually, reaching 29 articles by 2020. This study collected data on publications from 2020 to June 2024 through the Web of Science Core Collection and China National Knowledge Infrastructure (CNKI), finding a total of 275 published articles both domestically and internationally, averaging 55 articles per year. This demonstrates the growing importance of smart helmets. Figure 3 displays images of various smart helmets, and Figure 4 shows the keyword analysis of the 275 articles using CiteSpace.

CiteSpace provides two indicators, the modularity value (Q value) and the mean silhouette value (S value), based on the network structure of keywords and the clarity of clusters, to evaluate the effectiveness of the visualizations. A Q value greater than 0.3 indicates a significant community structure; when the S value is around 0.7, the clustering is highly efficient and convincing, and an S value above 0.5 is generally considered reasonable for clustering. The information panel in the upper left of the visualization provides various settings, such as the number of nodes, number of links, network density, silhouette values, and modularity values. As shown in the figures, the Q values for the two visualizations are 0.7468 and 0.655, both greater than 0.3, indicating significant clustering structures. The S values are 0.9404 and 0.8489, both greater than 0.7, suggesting that the clusters are reasonable. 

The number of publications on smart helmets is increasing annually, and the interest in smart helmets is clearly rising. In recent years, smart helmets in China have gradually expanded from military and transportation sectors to industrial monitoring, while abroad, the focus has been on upgrading smart helmets using artificial intelligence and deep learning methods. Notably, innovations in materials and comfort are also gaining attention. Considering the rapid development of flexible wearable devices in recent years, enhancing helmet comfort with flexible wearable sensors is a promising development direction. Table 1 summarizes the innovations in smart helmet development from other articles. The design features of smart helmets in the mining industry are generally similar [12,13,14,15,16,17,18,19], but there are also noteworthy innovations in non-mining industrial fields.

Overall, given the importance of helmets in the mining industry, smart helmet development has been rapid and comprehensive. Smart helmets monitor various human parameters such as body temperature, heart rate, EEG (electroencephalogram), fatigue level, respiratory rate, blood oxygen saturation, and personnel positioning. They also monitor environmental factors, including temperature, humidity, harmful gas concentrations, noise levels, light intensity, and collision avoidance in the surrounding environment. In addition to routine monitoring, current development directions for smart helmets include adding functions such as gesture recognition of operators, communication with surface units, detection of abnormal helmet usage, danger warnings, and automatic reporting of injury incidents [27]. In the transportation sector, features such as breath alcohol content detection and bone conduction technology in smart helmets are also promising for adaptation to mining smart helmets.

### 2.2. Smartwatch

The concept of smartwatches was proposed quite early, but due to technological limitations, true smartwatches did not start to appear until around 2010. In 2011, an Italian company was the first to produce a smartwatch that connected to a smartphone via Bluetooth [28], and by 2012, watches capable of independently running IOS and Android systems were developed [29]. Today, companies such as Apple, Xiaomi, Huawei, Samsung, Honor, and Fitbit [30] dominate over 60% of the market, with new products released annually and significant technological breakthroughs occurring every two years. Additionally, to reduce weight, lower costs, and improve usability, smart wristbands have emerged from smartwatches and have gained widespread popularity.

**Figure 5:**
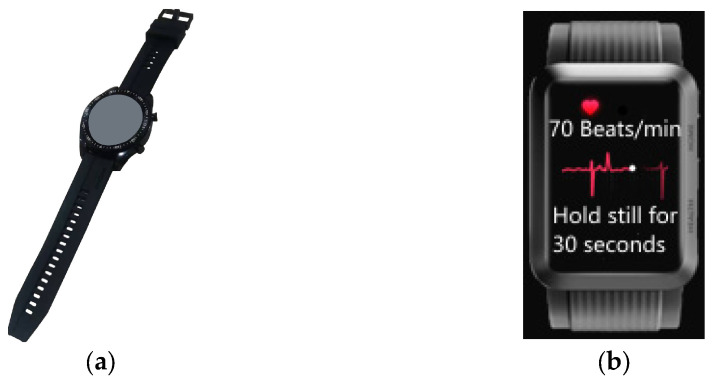
Smartwatch. (**a**) Smartwatch (coal safety type) [31]; (**b**) smartwatch heart rate test screenshot.

In the mining sector, smartwatches worn on the wrist greatly minimize restrictions on personal freedom while allowing for more accurate and convenient collection of physiological data from underground workers. A schematic of an intrinsically safe coal smartwatch is shown in Figure 5a, Beidou World Co., Ltd. Shandong branch, Jinan, Shandong, China, Figure 5b Chinese Institue of Coal Science, Beijing, China).

**Figure 6:**
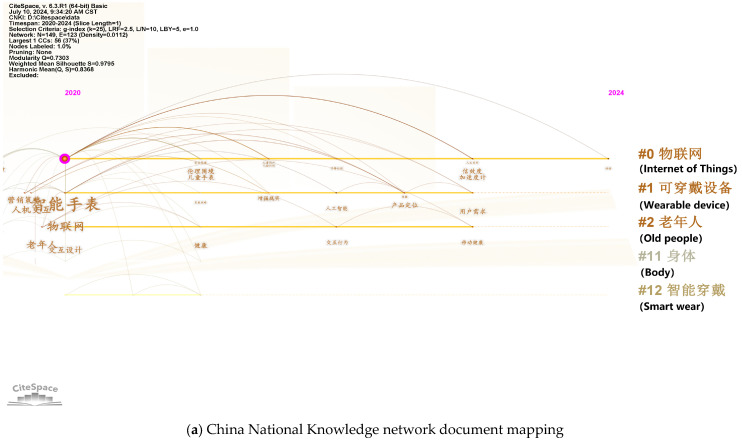

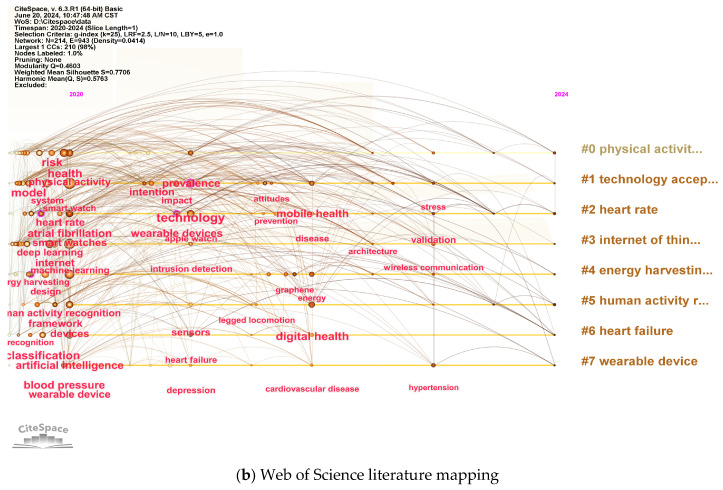
Plot according to the timeline with smartwatch as the keyword.

From 2020 to June 2024, a review of the Web of Science Core Collection and China National Knowledge Infrastructure (CNKI) revealed 492 articles published domestically and 713 internationally, averaging 241 articles per year. Using smart wristbands, a derivative of smartwatches, as a keyword search, CNKI returned a total of 833 articles, indicating that the trend toward lighter devices is a general development direction. Figure 6 shows the analysis of keywords from 1205 articles on smartwatches using CiteSpace.

The Q value for the CNKI visualization is 0.7307, and the S value is 0.9795, while the Web of Science visualization has a Q value of 0.4603 and an S value of 0.7706; these values all indicate reasonable clustering. The Web of Science keywords exhibit stronger associations, with most of the keywords aligning with the current directions of smartwatch development and focusing on upgrades, as well as building IoT systems centered around smartwatches.

Currently, sensors used in smartwatches can be categorized into three main types. The first type is physiological sensors, which include those for heart rate, body temperature, blood pressure, blood oxygen saturation, blood glucose, blood volume pulse, skin conductance, electrolytes, and ions. The second type is activity recognition sensors, which include motion gestures [32], body acceleration, proximity sensing of external objects, and pressure/strain sensors. The third type is environmental sensors, which monitor air temperature, altitude, light intensity, sound levels, atmospheric pressure, humidity, chemical gases, ultraviolet intensity, and personnel location [33].

Smartwatches are increasingly popular for health monitoring due to their small size, high performance, and high usability. However, the unique environment of underground operations necessitates a strictly customized design for smartwatches to ensure proper usage by workers. The characteristics of the underground smartwatch mainly include the following content: (1) The case needs to be resistant to a variety of chemical media corrosion, can maintain good mechanical properties in the high temperature environment of the coal mine and has good biocompatibility materials, such as titanium alloy. (2) The bottom cover is made of materials such as ceramics with low thermal conductivity, good insulation performance and does not affect the collection of human physical signs data. (3) Smartwatches need to collect a variety of human physiological indicators, such as heart rate, blood oxygen, etc., so they need to be equipped with a relatively good performance processor. (4) The battery adopts the anti-explosion treatment of the national standard polymer lithium-ion battery cell. (5) The underground network environment is complex, and the watch needs to support Bluetooth 5.0, WIFI dual-band connection or communication protocol adaptation and data transmission protocols of 4G/5G private networks in the coal mine as needed. Some examples of the development status of smartwatches are shown in Table 2.

In summary, the development of smartwatches in the mining industry continues to focus on enhancing the monitoring of personnel safety and health. Smartwatches provide monitoring functions for various physiological parameters, including body temperature, heart rate, EEG (electroencephalogram), fatigue levels, respiratory rate, blood oxygen saturation, and personnel positioning. Unlike helmets, which are essential equipment for underground operations, smartwatches offer a more compact option by occupying minimal space on the wrist. They also provide more accurate measurements of parameters such as heart rate and blood oxygen compared to smart helmets. Additionally, since many activities that impact health involve hand movements, wrist-based monitoring offers greater accuracy. Consequently, smartwatches are also designed to assess wearers’ conditions, such as smoking habits and work stress, by analyzing gestures and other wrist-based actions.

### 2.3. Other Intelligent Equipment

Smart helmets have led to the development of smart headbands, while smartwatches have evolved into smart wristbands and armbands. Aside from these devices, other smart equipment is gradually being introduced into mining operations, although their development is still not fully comprehensive. This section provides a brief overview of these advancements.

Since the concept of augmented reality (AR) was defined by Caudell and Mizell [43] in 1992, interest in smart glasses surged following the release of Google Glass in 2013. Smart glasses [44] are a type of optical head-mounted display that, in recent years, have integrated virtual reality (VR), augmented reality (AR), and Apple’s mediated reality (MR) technologies, overlaying virtual images onto the wearer’s view of the real world [45]. Despite variations in function and design, current smart glasses can generally be divided into two categories: the first type pairs with smartphones, requiring the display to be viewed on the phone screen, while the second type operates independently and can connect to external devices either wired or wirelessly.

According to a 2021 analysis by Dawon Kim [46] and colleagues, research publications related to smart glasses have been steadily increasing since 2014, with a slight decline in 2017, followed by a growth trend that surpassed previous years. In 2016, J. Jacobs [47] and others explored the potential applications of AR in the mining industry, concluding that AR could enhance worker productivity and reduce operational risks.

Initially, smart glasses were not primarily designed for industrial use, and many models were unsuitable for direct use in underground coal mining environments. To address this, ChenYu Zhang [48] and colleagues improved the design of smart glasses to enhance lighting performance, tailored to the low-light conditions typical of coal mines. Jieun Baek [49] and others developed a personnel proximity warning system for smart glasses, arguing that such wearable systems can improve work efficiency by freeing workers’ hands while ensuring safety. The proximity warning system enables smart glasses to receive signals from Bluetooth beacons attached to heavy equipment or vehicles, with the signal strength corresponding to proximity. Visual alerts are displayed on the glasses as the wearer approaches the equipment.

**Figure 7:**
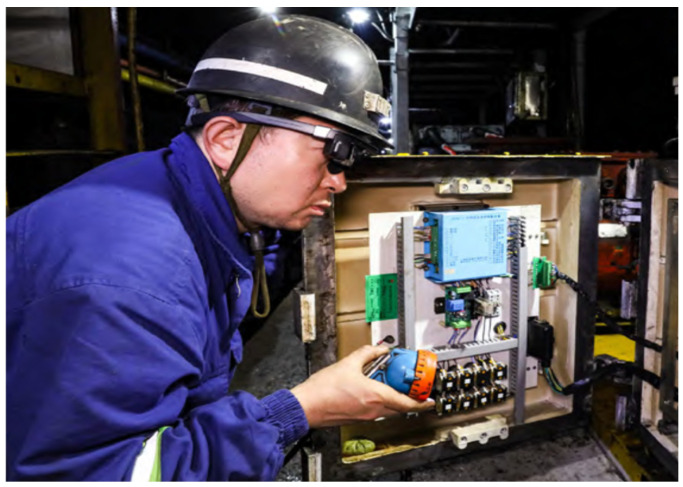
Application of smart glasses in mines [52].

In mining applications, smart glasses are often not standalone products but are used in conjunction with smart helmets. For example, Dawon Kim [50] and his team developed smart glasses that integrate with helmet-mounted devices, making them easier to wear, and used them with portable X-ray fluorescence analyzers to investigate soil contamination. Chen Chen [51] and others developed smart glasses that, paired with smart displays, assist with personnel tracking and diagnosing electronic equipment faults. These glasses can connect to a database, allowing users to access troubleshooting methods for equipment faults or request real-time assistance from remote experts. Figure 7 illustrates workers using smart glasses for standardized operational training.

The application of smart clothing also appears in mines with other devices. Roberto De Fazio et al. [53] designed a smart shirt that can monitor the heart rate, blood oxygen and temperature of the person wearing it. Subsequently, an electrochemical gas monitoring module was designed to obtain CO, O_2_, CH_2_O and H_2_S gas concentrations. The shirt has a battery life of about 16 days and is equipped with storage devices such as solar panels. Sakthivel Sankaran et al. [54] developed a smart jacket based on the smart shirt, and M. K. Chandrasekaran et al. [55] designed a smart jacket directly. It can mainly sense temperature, pressure, humidity and gas levels and other data, and the data will be displayed on the OLED display so that users can view it at any time. With the help of Internet of Things technology, all sensor data will be sent to the cloud. All supervisors need to do is install a mobile app that allows them to monitor the safety of all employees at all times. The smart clothing designed by Ming Li et al. [56] monitored the wearer’s health status by measuring electrocardiogram signals, and selected two optimal electrode positions for measuring ECG signals. The same jacket design is basically the same [57], with the main purpose of being able to monitor human data more comprehensively. For the high-temperature environment in underground coal mines, Yu Ma et al. [58], after comparing different cooling suits, concluded that air cooling suits are suitable for mines with a low pollution level, low cooling requirements, low humidity, and no electrical explosion-proof requirements. Liquid cooling suits are suitable for large spaces in high-temperature mines, and phase change cooling suits are suitable for short-term work in high-temperature mines. The selection of different cooling suits for different occasions can effectively reduce the surface temperature of coal miners.

**Figure 8:**
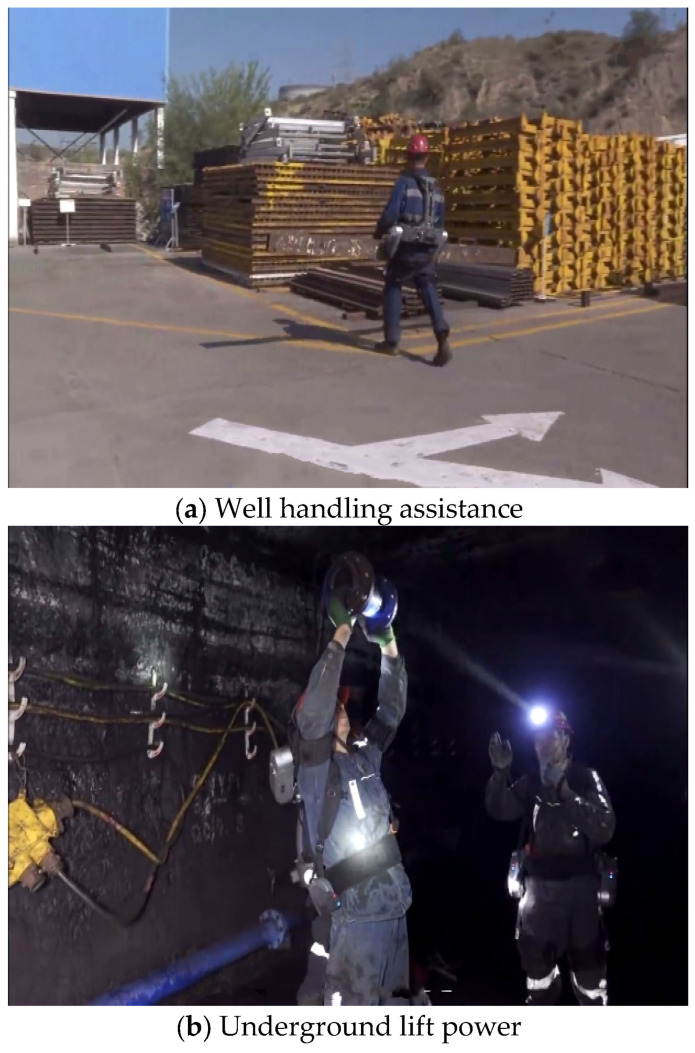
Application of exoskeleton equipment in mines [64].

In addition to traditional clothing, due to the fact that lifting heavy objects can lead to back sprains, muscle strains, and wrist, elbow, or spinal cord injuries, the development of skeletal support equipment in coal mines has been relatively promising in recent years [59]. Some mines are equipped with exoskeleton equipment for miners to help miners carry heavy objects, saving miners a lot of effort and reducing the risk of occupational diseases such as lumbar disc herniations among mining workers. Zefeng Yan et al. [60] proposed a wearable, lightweight exoskeleton that provides gravity support to workers to reduce the prevalence of musculoskeletal disorders, joint injuries, and arthritis caused by repeated or sustained squatting tasks. In their paper, Lu et al. [61] explained the research progress of using EMG to improve the intellectualization of exoskeleton robots. While effectively collecting EMG, the exoskeleton can follow actions through the EMG of miners, thus further guaranteeing the reduction of occupational diseases of miners. Kirsten Huysamen et al. [62] designed an exoskeleton to assist the worker in handling, helping the worker assist with hip extensor torque during lifting and lowering, reducing the musculoskeletal load on the lower back and keeping the contact pressure below the pain pressure threshold. Although exoskeletons have been gradually applied to the mining field, more studies are needed in terms of usability, such as explosion-proof testing and stiffness testing in coal mines, so as to ensure that exoskeletons can provide help during operation, rather than bringing new risks to operators [63]. The application of exoskeleton equipment above and below a well is shown in Figure 8 (ULS Robotics, Shanghai, China).

Smaller, discreet yet highly useful smart jewelry has also been developed to monitor human physiological health. L. Nivedhithaa and colleagues [65] designed a backpack for workers equipped with an oxygen supply and health monitoring capabilities. This device is made from fire-resistant materials and includes a mask with a heart rate sensor for oxygen intake, simultaneously monitoring the wearer’s heart rate. Rajan S [66] and his team developed wearable earmuffs to address the respiratory issues faced by miners underground. These earmuffs not only block harmful noise generated during mining but also prevent inhalation of toxic gases. The earmuffs feature internal fans and multi-layered masks to filter out dust particles. They are also equipped with humidity, temperature, and oxygen sensors to monitor various data parameters, ensuring the health and well-being of workers.

Since Oura launched its first smart ring in 2015, the company has released second and third generations in 2018 and 2021 [67], respectively. The third-generation ring includes an upgraded heart rate sensor, seven temperature sensors, and a new SpO2 sensor, providing a basic assessment of daily activity and rest duration. Notably, Oura’s smart rings do not use a single standard value for evaluations; instead, they establish a habitual baseline after a period of use, giving the wearer a personalized score for daily life. However, due to the ring’s small size and limited usable surface area, it must be paired with a smartphone for data viewing.

**Figure 9:**
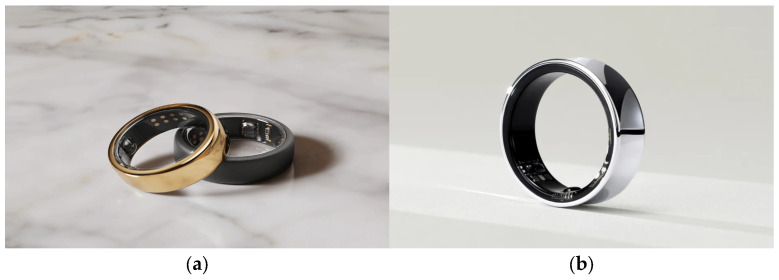
Smart ring display. (**a**) Third generation Oura ring [67]; (**b**) well handling assistance [68].

Samsung’s [68] upcoming smart ring is designed with the primary goal of monitoring sleep quality and is expected to track more physiological functions than Oura’s ring. However, such products are rare in underground coal mines and have only been tested in limited mining environments. An example of a smart ring is shown in Figure 9a Oura, Helsinki, Finland, Figure 9b Samsung, Suwon, Republic of Korea).

In summary, underground workers often endure long working hours in subterranean environments, and wearing large or heavy equipment for extended periods significantly increases their burden. Some research has focused on miniaturizing devices such as watches into bands or using jewelry like rings for monitoring vital signs. Although these studies are relatively limited, they have the potential to greatly reduce the load on workers during long shifts underground. By minimizing the weight burden on personnel, these innovations can also enhance the level of safety assurance for workers.

However, the development and application of smart equipment in the mining sector remains uneven. While many devices perform well in laboratory conditions, they may encounter operational issues or even fail to function when deployed in the field due to challenges such as the enclosed underground environment, dust, and poor signal transmission. Therefore, there is still significant potential for the further development and implementation of smart equipment in mining operations. On the whole, the smart helmets and smartwatches used in coal mines have made significant progress in technology, and their main key features are mainly reflected in safety monitoring, data transmission rate, wearing comfort and durability. However, due to the complexity of each coal mine environment and the uniqueness of each person, it is not possible to quantify some key parameters. Here, only some parameters for the unified development of wearable equipment in coal mines are given, mainly as follows: (1) Gas detection accuracy, about ±3–5% F.S. (2) Temperature monitoring range is about −20 °C to 50 °C, humidity monitoring range is about 0% to 95% RH. (3) Lightweight equipment weight, concentrated under 2 kg. (4) The data transmission distance is determined according to the complex environment of each mine, but the minimum effective transmission distance is generally kept above 30 m. (5) Battery capacity: most of the wearable devices currently use an independent power supply, which needs to maintain power for at least one working shift, and small devices such as watches should continue to work for about 1 week. (6) Portability and comfort, suitable for long-term wear; the material is usually silicone or other flexible, durable materials. (7) Protected performance, mainly including waterproof, fire resistance, seismic resistance, high temperature resistance, corrosion resistance, etc.

## 3. Indirect Monitoring of the Mine Environment

**Figure 10:**
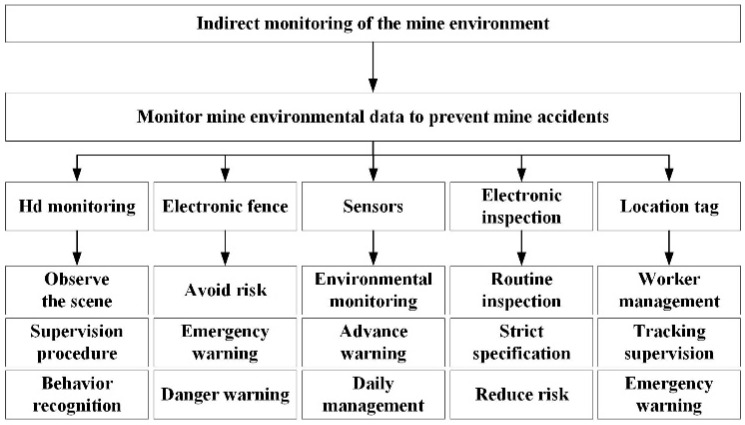
Indirect monitoring of the mine environment.

As the main source of China’s energy, coal has had a relatively complete mining system since a very early period. On the one hand, environmental monitoring of mines is the management of normal mining, and on the other hand, it also indirectly guarantees the safety of operators. Today, the mining field has formed a complete system to monitor and prevent the five major disasters of water, fire, gas, coal dust and roof collapse, supplemented by daily management of ventilation, preventing and controlling various safety hazards, and ensuring the safety of operators. By searching for the keywords of mine safety monitoring, mine disaster system, coal disaster system and coal safety on CNKI, about 2000 articles published after 2020 can be obtained, which shows the importance of mine safety. The way to ensure human health by monitoring the environment is shown in Figure 10 below.

**Figure 11:**
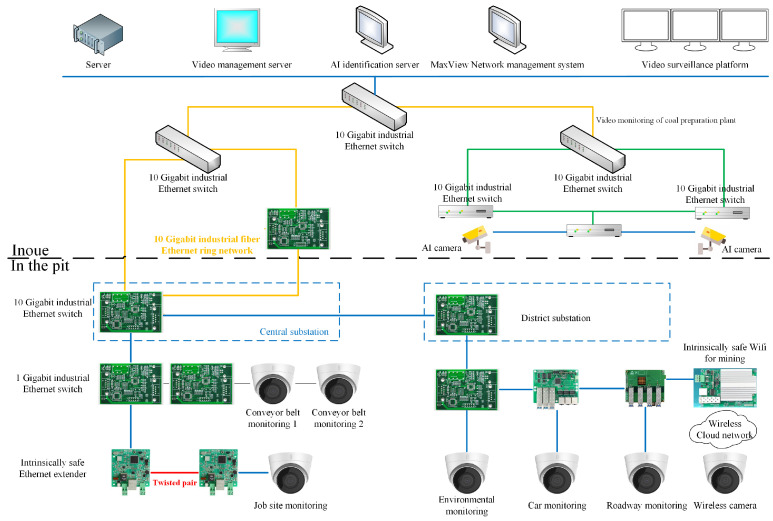
Mine video surveillance [71].

Figure 11 illustrates a mining video surveillance system that monitors environmental changes at the central control console using high-definition cameras. With ongoing technological advancements, traditional video and audio transmission-based monitoring methods are insufficient to ensure workers’ safety. Currently, camera technologies combine high-definition imaging with machine learning, such as using AI to assess the compliance of various work scenarios in terms of time and space dimensions [69]. This includes monitoring whether workers are wearing appropriate gear and assessing the surrounding environment. However, due to the complex mining environment, low visibility, and limited range, the selection of cameras must also consider critical parameters like shooting distance and explosion-proof design [70]. Thus, the application of video monitoring in mining remains a significant challenge.

Electronic fences have also emerged as a technology in the mining sector in recent years, primarily designed to prevent workers from accidentally entering hazardous areas, thereby avoiding injuries or fatalities [72]. Gansu Xigou Mining Co., Ltd. has implemented virtual electronic fences in critical mining zones, including loading, transport, and drilling areas, to ensure alarm systems, information transmission, incident management, and safety control in case of unauthorized access or equipment malfunctions during mining operations [73]. Additionally, the system can directly shut down equipment when the electronic fence triggers an alarm, further protecting workers’ lives [74]. Current electronic fences typically operate in a limited fashion, such as combining video surveillance with infrared beams or relying solely on tag recognition, which can lead to frequent false alarms. The equipment itself requires further development to ensure accurate and effective alerts.

Environmental monitoring sensors represent another crucial avenue for technological advancement in coal mining. Early mining sensors were relatively basic, often limited to temperature and humidity data, but an increasing variety of sensors are now being used. Ventilation monitoring, a critical aspect of mining safety, requires numerous sensors to collect data. Common sensors in domestic coal mine monitoring systems include those for gases, temperature, coal stock levels, water levels, and voltage [75]. Ventilation monitoring is especially vital for ensuring safe breathing conditions and preventing mining disasters like explosions. Key monitored gases include O_2_, CO, CO_2_, CH_4_, SO_2_, H_2_S, and wind speed. T. Liu et al. [76] have focused on deploying fiber optic sensors in coal mines to monitor roof integrity and hazardous gases. Tests in certain mines have shown that fiber optic methane sensors offer advantages over conventional electrochemical methane sensors, such as not requiring routine calibration. Field tests with fiber optic microseismic sensors have also proven their utility. Due to their anti-interference, corrosion resistance, and high sensitivity, fiber optic sensors have become a primary choice for mine environmental monitoring in recent years [77]. Overall, while these sensors primarily monitor the mining environment, their main goal remains to ensure a safe working environment for personnel.

**Figure 12:**
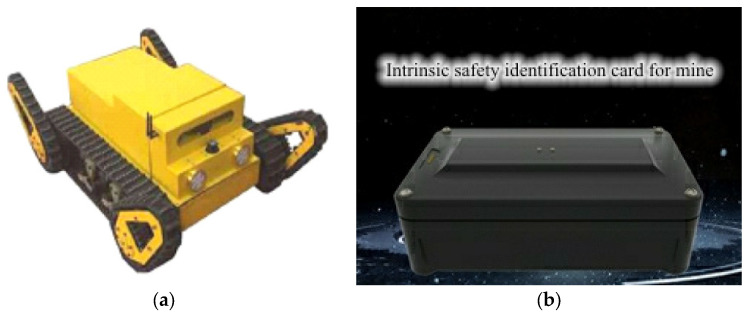
Monitoring equipment. (**a**) Crawler inspection robot [83]; (**b**) location identification card [84].

With the rise of smart mines in recent years, electronic inspection robots and worker positioning tags have undergone continuous improvements and upgrades. Electronic inspection robots replace workers in conducting inspections, significantly reducing their exposure to dangerous areas and thus enhancing safety [78]. The use of these robots also improves the accuracy of environmental monitoring in mines, reduces human errors, and increases efficiency in inspections and fault handling [79,80,81]. Figure 12a shows an inspection robot. Worker positioning tags are used to precisely track workers’ locations and spatiotemporal distribution in complex tunnel environments, enabling the central control station and management to monitor underground movement patterns in real-time. This provides a basis for efficient mine scheduling, emergency command, and scientific rescue efforts, as illustrated in Figure 12b [82].

## 4. Research on Internet of Things Assisted Decision-Making in Mines

Connecting multiple devices through wireless or wired networks to build an integrated mining IoT system, centrally managed at the main control console, enables a comprehensive monitoring system that combines environmental and vital sign data, providing a robust safeguard for workers’ safety. Specifically, IoT for wearable devices can connect all wearable sensors, process and analyze the data in the cloud, and then send it back to the wearables, achieving significant advancements in efficiency, memory usage, and device size optimization. The continuous monitoring of mining conditions generates a vast database, and wireless data transmission can optimize the efficiency of data processing and transfer. As an intermediary hub between wearable devices and IoT servers, wireless networks facilitate real-time, bidirectional communication between individual workers, smart equipment, and the main control console, as well as between mining environment data and the control center.

In recent years, various sensors have been deployed across diverse fields, making IoT a hot topic for connecting everything and assisting users in smart decision-making. However, the complexity of the mining environment poses significant challenges to upgrading underground internet infrastructure [85,86]. As early as 2007, Sammar et al. [87] discussed the use of intelligent wireless sensor technologies in hazardous mining environments, highlighting the indispensability of wireless network technology as a bridge connecting smart equipment. Hong Cong Nguyen et al. [88] recognized the potential of wearable devices in mining, and focused on a star-shaped wearable wireless sensor network system for remote monitoring of miners’ health and environmental parameters using low-power Bluetooth and Wi-Fi technologies. This system facilitates data transfer to the main control console for monitoring and alerting workers. Lalatendu Muduli et al. [89] reviewed literature up to 2018 on using wireless sensor networks for online monitoring of underground coal mines, identifying interdisciplinary studies that applied these networks for environmental monitoring, personnel tracking, and smart safety with underground tags.

Overall, wireless networks connect various elements to form an integrated smart mining system. Yuchun Zhang et al. [90] argued that establishing a wireless local area network (WLAN) is critical for real-time data collection and analysis, comparing solutions and finding that the ETPA, TARA, and LTRT protocols are suitable for building underground WLANs.

While wireless networks offer convenience, their performance is often suboptimal in underground environments, making wired networks essential in certain scenarios [91]. Key considerations when choosing between these technologies include interference resistance, operational stability, reliability, explosion-proof performance, and transmission range. However, the main drawback of wired networks is their vulnerability to damage from underground conditions, such as cable breakage or disconnection, which can be common due to the ever-changing nature of mining sites [92]. Additionally, the extensive deployment of cables over long distances significantly increases the workload in underground operations.

**Figure 13:**
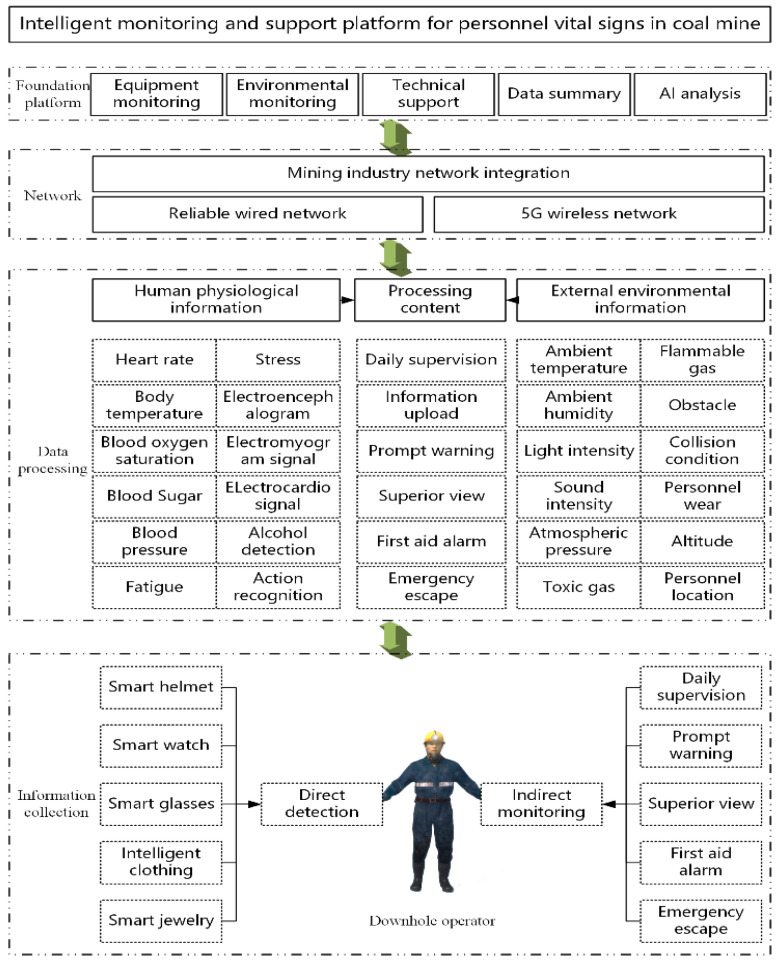
Intelligent monitoring and support platform for personnel vital signs in coal mines.

To ensure reliable network performance underground, a hybrid approach combining wired and wireless networks is commonly employed for data transmission. Beyond this, Augustus E. Ibhaze et al. [93] compared various downhole communication methods used in the oil and gas industries, noting that some are applicable to coal mines. In summary, whether dealing with physiological information or mining environment data, there is always a latency in data aggregation [94]. Ensuring that this latency remains within acceptable limits is a critical consideration when integrating wireless and wired networks. This integration culminates in the comprehensive platform workflow illustrated in Figure 13.

The mining environment is complex and highly hazardous, making traditional management and decision-making approaches inadequate for meeting modern safety and efficiency demands. With the rapid advancement of information technology, particularly in artificial intelligence, big data, and the Internet of Things (IoT), mining decision support systems are becoming essential tools for enhancing management and safety in mining operations. Developed countries in Europe and North America have already implemented advanced technologies such as cloud computing, IoT, and AI in mining. These technologies enable precise management of mining operations through intelligent monitoring systems and decision support platforms, significantly improving productivity and safety. In contrast, domestic mining often employs sensors, wearable devices, and wireless communication technologies to build separate systems for environmental monitoring, personnel tracking, and production data collection. These systems can monitor environmental parameters such as gas concentration, temperature, and humidity in real-time, and manage the mining environment, equipment status, and personnel safety. However, only a few coal mines have integrated these systems into a unified, big data-driven intelligent decision support system.

Independent systems include the direct use of AI or machine learning algorithms and other content, so that individual equipment quickly achieves early warning or alarm function. Ali [95] concluded in 2020 that in the mining sector, machine learning and artificial intelligence can be applied from the beginning of mining to the end of the mine life cycle, from exploration to production to closure and mine reclamation. However, unlike other industries, the mining and metals sector is considered to have a lower digital utilization rate. Intelligent monitoring of vital signs in coal mines requires a lot of AI or machine learning algorithms. In the complex environment of underground coal mines, the accuracy of smart sensors used to monitor vital signs such as temperature, heart rate and respiratory rate is often slightly lower than in the laboratory, or even just in the well. For example, due to environmental temperature, humidity, or downhole dust, electromagnetic interference and other factors, the data collected by the sensor often contains a large number of errors, and when the accuracy of the sensor is below a certain extent, higher precision equipment cannot greatly optimize the data, and it also increases the cost. Therefore, how to ensure the reliability of the collected data under the premise of ensuring the light weight and comfort of the equipment has become a platform for AI and machine learning to play a big role. For example, the factors influencing wearable devices often come from the human body itself or from complex external magnetic fields, such as the acquisition of electromyography signals, often due to a high underground temperature resulting in a large amount of sweat of operators, or long-term muscle fatigue resulting in the accuracy of the acquisition of electromyography signals over time getting lower and lower. H. Pan [96] designed a method for fatigue state detection based on a multi-modal extraction fusion framework. From the collected electrocardiogram, skin electrical activity, blood pressure, blood oxygen saturation, skin temperature and other physiological data, as well as facial data, a multi-modal feature extraction fusion framework is used, and Transformer+ multi-feature fusion is used to present a fatigue state. It can also be found that the acquisition of a certain parameter is often not a simple one-to-one, but many-to-one, and a variety of sensors jointly collect the same parameter, thereby reducing the unreliability of the data.

Traditional underground coal mine environments are often covered with coal dust, and although the space is narrow, ordinary cameras often exhibit low visibility and a wide range of blind areas. In some studies, active learning algorithms are used to train on the scene situation collected by the camera, so as to reflect the scene situation more clearly. Taking anti-collision in coal mines as an example, Mohamed Imam [97] introduced RGB cameras, infrared cameras, stereoscopic cameras, light detection and ranging to carry out more comprehensive video surveillance using four modes: UAV, electronic inspection vehicles, mobile machinery in the mine and cameras with a fixed position. Among them, the mobile carrying method ensures its normal work through the machine learning method in the early stage, such as the training of the UAV through a convolutional neural network to autonomously pass through a dark underground mine [98]. A coal mine rescue robot has the ability of autonomous obstacle avoidance when conducting environmental detection and rescue through accurate three-dimensional reconstruction training of the on-site environment [99].

How to combine the collected physiological data, environmental data, video surveillance and other data has become another important research direction. There are two main ways to relate data to the actual situation, namely model methods and non-base model methods [100]. In this paper, the model method means to establish a model between physiological data, environmental data and miners’ vital signs, and obtain the relationship between the mine environment and miners’ vital signs according to the linkage model. This method can accurately establish the mapping relationship between signals and the human body, but it requires complex parameter calibration. A non-basic model method directly establishes the mapping relationship between physiological data, environmental data and miners’ vital signs by using machine learning and deep learning algorithms. Usually, from the initial discrete data classification, physiological features of offline data are identified based on the extracted features. After the obtained features are labeled and classified, methods such as a majority voting mechanism are used to determine the possible impact of a class of data features.

Integrating these independent systems can create a comprehensive decision support platform through data collection, storage, and analysis, providing scientific decision-making support for managers. This platform can optimize production plans, improve equipment maintenance strategies, and ensure a safe working environment, thereby enhancing productivity and resource utilization. As wearable devices become more widespread, they facilitate diverse physiological data collection, which, when combined with machine learning, AI, and big data models, can aid in decision-making at the main control center. For example, when workers wear AR glasses, the glasses can display standardized operational procedures and immediately correct any errors, preventing unnecessary injuries due to negligence. Moreover, if underground workers experience abnormal physiological changes, supervisors can promptly receive alerts and dispatch personnel to assist, gathering real-time information about the site. For equipment maintenance, regular security inspection is generally carried out by personnel, but the occurrence of problems is often a matter of an instant, and regular inspection becomes less reliable. Through the system platform, it is easiest to monitor the data change curve of each piece of equipment and simply analyze whether the data error is large, so as to determine whether the equipment needs maintenance, or based on the frequency of regional use, the maintenance time of the equipment can be dynamically improved.

Qing Dong et al. [101] evaluated the use of multi-objective wireless sensor detection for assisting in engineering risk management of mining geological environments. By vectorizing various evaluation factors and applying a fuzzy comprehensive evaluation method, they assessed the geological environment of a high-top mountain mine, providing theoretical support for mine environment restoration and management research in China. Xiao Wang et al. [102] developed a coal mine safety risk intelligent management and information decision analysis system, which identifies risk sources in different spatial and temporal dimensions of safety production. This system breaks down the coal production system into independent evaluation factors, overcoming the variability and complexity of the natural environment and associated risks. By summarizing the main factors influencing safety production risks and determining weights using qualitative and quantitative methods, they constructed a risk evaluation index system for coal mine safety production. The system’s accuracy was validated through three field tests.

Some mining enterprises focus on the system’s emergency response capabilities. Intelligent decision support systems enable simulation and predictive analysis to pre-emptively devise emergency response plans for sudden environmental changes or emergencies. These systems can suggest optimal solutions to assist the main control console in making decisions, such as broadcasting emergency evacuation instructions, mapping optimal escape routes, and coordinating with military or medical assistance to minimize accidents and losses and reduce rescue times. Thus, when reliable data acquisition is assured, mining decision support systems become indispensable for avoiding human error and ensuring safe production activities. For instance, Alexander Smirnov et al. [103] documented decision-makers’ preferences in emergency response systems in a database, accumulating extensive records of current situations, user preferences, proposed solutions, and final decisions.

In addition, the situation of each mining area is often different. In summary, the cost of building sensors, wearable devices and platforms is often a high consumer cost. Large mining areas have sufficient funds and better conditions to ensure the basic safety of personnel. However, small mining areas and mining areas with relatively low income are often unable to carry out excellent platform construction from the perspective of capital and long-term benefits. In contrast, such mining areas can directly use specific intelligent monitoring and support equipment for the vital signs of coal mine personnel, without the need to integrate a large platform. According to the characteristics of each mining area, the selection of specific equipment can directly reduce the cost and guarantee the life, health and safety of operators.

Overall, while individual smart devices can measure various data, they are not designed to fully consider all possible data sources. Deciding on the best device for each type of data based on the optimal measurement location and accuracy is crucial for maximizing the benefits of using multiple smart devices together. Therefore, integrating and coordinating all data through wireless or wired networks and processing it at the main control console is essential for maximizing worker safety [104]. Given precise big data collection, using big data models, machine learning, and AI can effectively assist leaders and field personnel in decision-making. However, as technology advances, new challenges emerge. IoT-based wearable devices contain confidential and sensitive information about workers. Much of this data relates not only to the miners’ physical information but also to the basic conditions of the mine, making it highly sensitive. Therefore, data security and privacy are paramount. In the medical field, where wearable device development is more advanced, privacy protection of patient data is a key concern. Ke Wang et al. [105] designed a ciphertext-policy attribute-based encryption for IoT, which ensures that data access is granted only when the data user’s attributes match the access policy set by the data owner, protecting patient information without increasing the system’s response time.

## 5. Summary and Prospects

This paper provides a detailed overview of the research progress in intelligent monitoring and protection of miners’ vital signs in underground coal mines, analyzing the application and development trends of current technological methods. The focus is primarily on two well-developed devices: smart helmets and smartwatches, while also briefly addressing the development and use of other intelligent equipment. In the mining industry, the direct monitoring of miners’ vital signs using intelligent devices and the indirect monitoring of environmental parameters through sensors installed in the mines are both crucial strategies for providing comprehensive safety protection for miners. This article further explores the key connecting technology for integrating these two approaches—the Internet of Things (IoT)—and its significant role in enhancing coal mine safety. In the future, as big data and artificial intelligence technologies continue to advance, vital sign monitoring will become more intelligent and personalized. By comparing and analyzing historical data, it will be possible to more accurately predict and assess miners’ health status, providing personalized health management solutions for the workforce.

However, the current development of mining intelligence is still uneven, insufficient, and uncoordinated. On the one hand, intelligent equipment for safeguarding the lives of underground coal miners is relatively limited. While independent devices have seen significant progress, a comprehensive system that integrates these devices to collectively ensure the safety of miners is not yet fully developed. Each device only provides limited protection, and to fully safeguard all vital signs of underground miners, multiple devices need to be worn, which can increase the physical burden on the workers. On the other hand, issues related to data privacy and security also require more research and innovation to ensure the protection of miners’ personal information.

Therefore, this paper presents the following outlooks on the intelligent monitoring and protection of underground miners’ vital signs:

1. Development of Integrated Monitoring Devices for Environmental and Vital Sign Data. While existing devices demonstrate proficiency in monitoring either environmental or vital sign parameters independently, the development of systems capable of integrated monitoring remains underexplored. Smart helmets currently lead in combining these functions, but they are often bulky and impractical for prolonged use in underground mining. Miniaturized wearables, such as wristbands and rings, have shown potential for reducing miners’ physical burdens while maintaining essential monitoring functions. Future research should prioritize the design and development of lightweight, compact, and multifunctional wearable technologies. Such advancements could integrate both environmental and physiological monitoring capabilities into a single device or interconnected system, ensuring comprehensive safety while enhancing user comfort and reducing fatigue.
2. Advancement and Application of Intelligent Decision Support Technologies. The transition to intelligent mining, propelled by AI and machine learning innovations, underscores the need for smarter decision-making systems. Current efforts focus on improving the intelligence and automation of mining equipment, enabling cross-platform integration, and enhancing data sharing. These developments aim to optimize emergency responses, predictive maintenance, and safety management. Future research could delve deeper into real-time adaptive decision-making frameworks, advanced predictive analytics for equipment lifecycle management, and AI-driven models tailored to complex mining scenarios. The integration of environmental sustainability metrics into these systems is another promising area, aligning technological advancements with ecological considerations.
3. Privacy and Security in Big Data Applications. As data collection becomes more pervasive in mining operations, safeguarding the privacy and security of sensitive information is paramount. Data used to monitor miners’ health and safety also often includes proprietary information about mining operations, making it a target for potential misuse. Future research should explore robust encryption methods, secure data-sharing protocols, and advanced intrusion detection systems to protect this data. Additionally, developing frameworks for compliance with global data protection standards and exploring AI-driven anomaly detection for real-time security monitoring are critical directions. Ensuring a balance between data utility and privacy will be essential to maintaining trust and protecting both individual rights and commercial interests.

Researching the patterns of changes in physiological indicators from the perspective of safety production and health protection is of great practical value in accident prevention, injury reduction, and the protection of occupational health. Only by ensuring the health and safety of the workforce can underground production activities be carried out safely and efficiently. Through ongoing technological innovation and application, the aim is to further enhance the safety and intelligence of coal mine production, ultimately ensuring the health and well-being of underground miners.

## Figures and Tables

**Table 1:** Innovative development of smart helmets.

Author	Domain	Innovation
Yeanjae Kim et al. [20]	Mining industry	Add an approach warning system for those wearing smart helmets to prevent collisions between equipment and pedestrians in mines.
Yiyang Zhuang et al. [21]	Mining industry	Embedded fiber-optic sensors directly into the thin surface layer of the helmet housing to enable the device to sense and analyze blunt force impact events that could provide a direct way to assess the helmet’s head protection capabilities and assess the potential physiological effects of blunt force impact events in real time.
Lalitha K et al. [22]	Mining industry	A convolutional neural network algorithm was used to train an AI model to recognize workers’ gestures to help operators communicate. Gestures are labeled “good”, “bad”, “doing well”, and “emergency evacuation”.
Lei Wang et al. [23]	Mining industry	Integration of wireless communication technology, positioning technology, and a sound and light alarm module. The ZigBee terminal node is used to upload the data to the gateway, and the location information of the staff is determined according to RSS positioning technology to provide the corresponding emergency treatment.
Lili Kong et al. [24]	Mining industry	The traditional helmet is equipped with toxic gas monitoring, temperature and humidity and other environmental information collection sensors, and pressure monitoring is used for early warning of abnormal situations, and the mine working environment information is actually monitored.
A. Jesudoss et al. [25]	Transportation field	Equipped with infrared sensors to check if the person is wearing a helmet and a gas sensor that identifies alcohol in the rider’s breath.
Yanseng Li et al. [26]	Transportation field	By using modules such as WTGPS+BD Beidou positioning and bone conduction Bluetooth technology in the smart helmet, navigation and positioning, Bluetooth broadcasting, LCD display, pulse heartbeat monitoring, automatic alarm and other functions are realized.

**Table 2:** Innovative development of smartwatches.

Author	Domain	Innovation
Sayan Sarkar et al. [34]	Mining industry	Smartwatch designed to monitor and analyze different cardiorespiratory and humoral based parameters of downhole operators, recording performance indicators and stress of operators.
W. Pratt Rogers et al. [35]	Mining industry	With existing smartwatches, it is possible to assess different fatigue conditions for downhole operators by combining the ability to evaluate performance measures in real time with subjective measures.
Yufeng Jiang et al. [36]	Mining industry	On the basis of non-invasive measurement to avoid environmental interference and ensure the explosion-proof requirements, a bracelet with high integration and lightweight structure was designed for monitoring the blood oxygen saturation, body temperature and pulse wave data of the human body.
Alessandro Leone et al. [37]	Industry	Using a combination of wristbands and EOGs, a framework was designed to analyze heart rate, electrodermal response, and EOG signals to extract features that could monitor excessive stress or cognitive load.
SA Khowaja et al. [38]	Industry	For the purpose of a low cost and low sampling rate, a smartwatch was designed, and the monitoring performance was improved by the method of machine learning, and the stress can be detected in the real environment.
W Sanchez et al. [39]	Industry	Compare multiple machine learning algorithms to identify work stress in its initial stages through the behavior and physical activity of employees as monitored by Fitbit^®^ wrist-worn sensors.
Víctor González et al. [40]	Industry	Applied a variety of metal oxide sensors to achieve the use of watches for toluene, xylene, and ethylbenzene gas detection.
Abhinav Parate et al. [41]	Daily	Methods of detecting gesture behavior with a watch were designed to judge the eating and smoking situation of watch wearers from their usual gestures.
ANDUN et al. [42]	Medical	Through long-term monitoring of human health, sleep health, psychological pressure, blood pressure, blood sugar, blood lipids and other data, the self-developed algorithm is used to predict the occurrence probability of more than 10 diseases and chronic diseases in the next 30 days.

## Data Availability

The raw data supporting the conclusions of this article will be made available by the authors on request.
